# Left atrial spatial entropy: a novel tool for electrophysiological substrate characterization in atrial fibrillation

**DOI:** 10.3389/fphys.2024.1474568

**Published:** 2024-11-27

**Authors:** Lorenzo Gigli, Alberto Preda, Davide Coluzzi, Marta Sartore, Muhamed Vila, Marco Carbonaro, Matteo Baroni, Marisa Varrenti, Sara Vargiu, Fabrizio Guarracini, Antonio Frontera, Luigi Pannone, Gian Battista Chierchia, Carlo De Asmundis, Patrizio Mazzone, Roberto Sassi

**Affiliations:** ^1^ De Gasperis Cardio Center, Electrophysiology Unit, Niguarda Hospital, Milan, Italy; ^2^ Heart Rhythm Management Centre, Postgraduate Program in Cardiac Electrophysiology and Pacing, Universitair Ziekenhuis Brussel – Vrije Universiteit Brussel, Brussels, Belgium; ^3^ Department of Computer Science, University of Milan, Milan, Italy

**Keywords:** atrial fibrillation, left atrial spatial entropy, catheter ablation, substrate characterization, mapping technologies

## Abstract

**Background:**

Electrical remodeling has been linked to the progression and recurrence of atrial fibrillation (AF) after catheter ablation (CA). Substrate mapping based solely on a voltage amplitude electrogram (EGM) does not provide a comprehensive understanding of the left atrial (LA) disease. The aim of this study is to assess left atrial spatial entropy (LASE) from voltage maps routinely obtained during AF ablation to further characterize the LA substrate.

**Materials and Methods:**

High-density electroanatomic maps (EAMs) of 27 patients with paroxysmal or persistent AF undergoing routine CA were prospectively collected. Computational post-processing was performed on the voltage maps. Using the Shannon entropy model, the probability distribution of the amplitude range values associated with each point of the map was used to measure LASE. Finally, correlations between LASE and clinical and electrophysiological characteristics of AF were explored.

**Results:**

LASE differentiated between patients with paroxysmal and persistent AF (6.45 ± 0.41 vs. 5.87 ± 0.53; *p* = 0.028) and patients with normal and abnormal LA substrate (6.42 ± 0.42 vs. 5.87 ± 0.56; *p* = 0.043), independent of the basal rhythm during EM acquisition (6.33 ± 0.41 vs. 6.11 ± 0.63; *p* = 0.619). Accordance between LASE and EAMs was assessed by ROC analysis (AUC: 0.81; C.I.: 0.62–0.99; Youden index: 6.06; sensitivity: 80%; and specificity: 80%). Patients with the lowest LASE reported AF recurrence at the follow-up.

**Conclusion:**

LASE may play a role in the further characterization of the LA substrate and the type of AF, independent of basal rhythm.

## Graphical Abstract

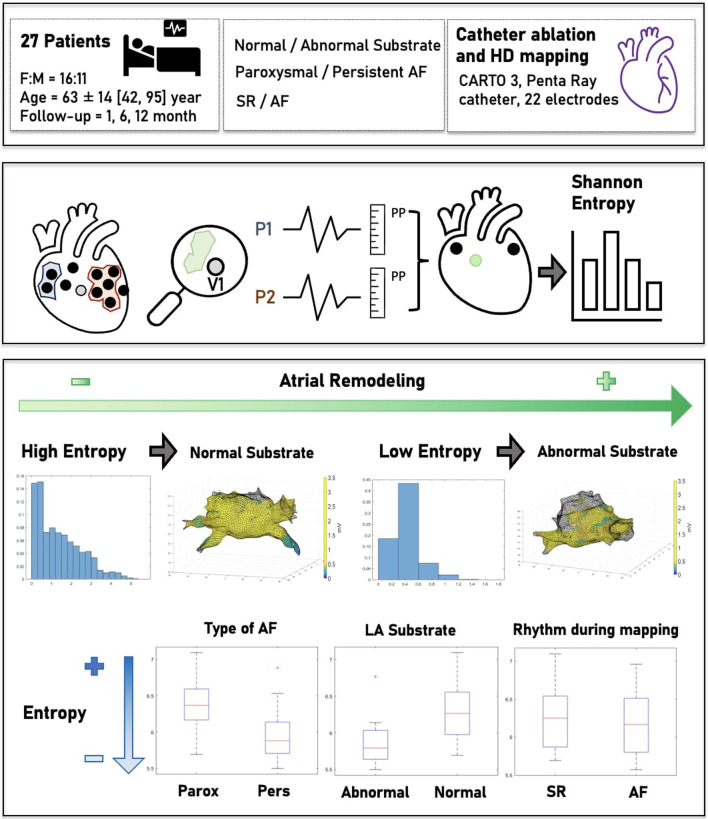

## 1 Introduction

Atrial fibrillation (AF) is the most common arrhythmia worldwide, associated with increased morbidity and mortality (Chugh et al., 2014). Sinus rhythm (SR) maintenance is the main target of AF treatment, and catheter ablation (CA) is currently the gold standard used to prevent arrhythmia recurrence (Arbelo et al., 2014). Continuous technological advances in the characterization of anatomical and electrical substrates, as well as in ablation methods, have allowed us to better understand AF mechanisms and improve outcomes (Parameswaran et al., 2021). However, the prediction of further events after CA is still a matter of debate (Berruezo et al., 2007; Li et al., 2023; Marcon et al., 2023). During bipolar mapping, the identification of low-voltage areas (LVAs) using universally accepted thresholds is considered proof of cardiomyocyte disease and left atrial (LA) fibrosis (Rodríguez-Mañero et al., 2018), which is associated with the advanced stage of AF and, consequently, a higher risk of recurrence (Vlachos et al., 2017). However, voltage mapping, based on electrogram (EGM) acquisition, has some well-known limitations, mainly due to the variability in signal morphology according to the wavefront direction with respect to the electrode position (de Bakker, 2019), electrode length, interelectrode spacing, and tissue contact (Yamaguchi et al., 2019). Finally, the heart rhythm highly influences EGM acquisition and, thus, its interpretation (Jadidi et al., 2016).

Entropy was introduced in thermodynamics as a measure of randomness and “disorder” in a molecular system. Its application has since expanded across numerous scientific domains, including information theory, data mining, and mathematical linguistics (Shaw and Charles, 1983). In the medical field, entropy was largely used to evaluate the state of consciousness (Mateos et al., 2018) and analyze the homogeneity of the heart tissue and its relationship with cardiac events (Wu and Chrispin, 2022).

In particular, the measurement of entropy from a distribution of signals (called “Shannon entropy”) and its links to the probability theory, as described in the information theory, was applied to cardiac electrophysiology to differentiate complex fractionated atrial electrograms (CFAEs) in patients with AF from background noise (Ng et al., 2010) and distinguish bipolar EGMs recorded at pivot points of rotors from EGMs detected in peripheral regions (Ganesan et al., 2013). Hwang et al. (2016) highlighted the potential use of Shannon entropy to evaluate the nature of rotors in 2D and 3D *in silico* models of persistent AF. To date, despite its potential relevance in this field, no studies have applied and evaluated entropy directly computed from EGM-based voltage maps across diverse cohorts of AF patients.

The aim of this study is thus to quantify left atrial spatial entropy (LASE) for further characterizing LA tissue in paroxysmal AF (paAF) and persistent AF (peAF) patients. We applied the Shannon entropy model to process a whole mass of data from voltage maps routinely collected during AF ablation.

## 2 Materials and methods

### 2.1 Study design

The data from 27 consecutive patients with either paAF or peAF admitted to the electrophysiology unit of Niguarda Hospital, Milan, Italy, in January 2022, who underwent CA of AF, were prospectively collected and analyzed. The study was conducted in collaboration with the department of Computer Science of the University of Milan, Italy. Inclusion criteria were as follows: patients aged >18 years with paAF or peAF who had a first indication for AF ablation. Exclusion criteria were as follows: the presence of LA appendage thrombus at pre-operatory imaging, previous ablations of any type, and the need for additional ablations (i.e., cavotricuspid isthmus isolation). Baseline clinical characteristics were recorded through digital medical reports. Electroanatomic maps of LA were obtained using the CARTO 3 electroanatomic mapping system (Biosense Webster, Diamond Bar, CA, United States). For each patient undergoing AF ablation, a bipolar, high-density map of the LA was obtained. The resulting data, comprising the spatial position of the catheters, electrical signals, and vertices and triangles of the LA mesh, were extracted, exported, and analyzed offline. After discharge, patients were followed up with 24-hour Holter monitoring and clinical examinations performed at 3, 6, and 12 months. The primary endpoint was to compare the LASE with the AF-type and LA electroanatomical map (EAM).

The study was approved by the Ethics Committee of Niguarda Hospital in compliance with the Declaration of Helsinki. All patients provided informed consent before the procedure.

### 2.2 Mapping and ablation procedure

All procedures were performed under general anesthesia by an electrophysiologist experienced in AF ablation, assisted by at least one additional electrophysiologist or fellow. The whole CA methodology has been previously described in the work by Pratola et al. (2008). A deflectable decapolar catheter (Bard Dynamic) (Boston Scientific, Marlborough, MA, United States) was positioned in the coronary sinus. A single trans-septal puncture was performed with the standard electrophysiological approach. After a trans-septal puncture, intravenous heparin was administered to maintain an activated clotting time of >300 s. Real-time 3D LA maps were reconstructed using the PentaRay mapping catheter (Biosense Webster, Diamond Bar, CA, United States) with a minimum of 2,000 anatomical points acquired. The maps were acquired independently in the presence of SR or AF, and the normal LA substrate was distinguished from the abnormal LA substrate according to universally accepted voltage thresholds, setting low and high band pass thresholds at <0.05 mV and >0.5 mV, respectively (Vlachos et al., 2017). In particular, the LVA was defined by ≥ 5 adjacent points of <0.5 mV acquired during SR and using a multipolar HD catheter. The accuracy of point acquisition was evaluated through the Tissue Proximity Index (TPI) algorithm using the CARTO 3 system to limit low-contact artifacts. Furthermore, multiple local remaps with a minimum density of ≥10 points were performed to reduce wavefront direction distortion. The entire mapped LA was generally as defined abnormal by >1 LVAs adjacent or localized in different LA regions. In addition, to reduce the acquisition of false positive LVAs due to inadequate contact with the wall, all EAMs were acquired using the activated Tissue Proximity Indicator (TPI) tool of PentaRay. Anatomic reconstruction of the LA, including the LA–pulmonary vein (PV) junction, was performed. In all patients, radiofrequency (RF) ablation was performed to create a single circumferential line around contiguous veins. RF pulses were delivered with a temperature setting of up to 43°C and 35 W using the THERMOCOOL SMARTTOUCH SF Catheter (Biosense Webster, Diamond Bar, CA, United States) targeting an ablation index (AI) of 500 for both the anterior wall and roof and 400 for the posterior wall and floor of LA. The ablation lines consisted of contiguous local lesions <5 mm from each other and at a distance of >5 mm from the ostia of the PVs using the wide antral circumferential ablation technique (Proietti et al., 2014). In cases of LVAs on the LA posterior wall, posterior box isolation was performed (Romero et al., 2023). Procedural success was defined according to universally accepted criteria for pulmonary vein isolation (PVI) and LA posterior war isolation by checking entrance and exit blocks (Tzeis et al., 2024).

### 2.3 Data acquisition and processing

**FIGURE 1:**
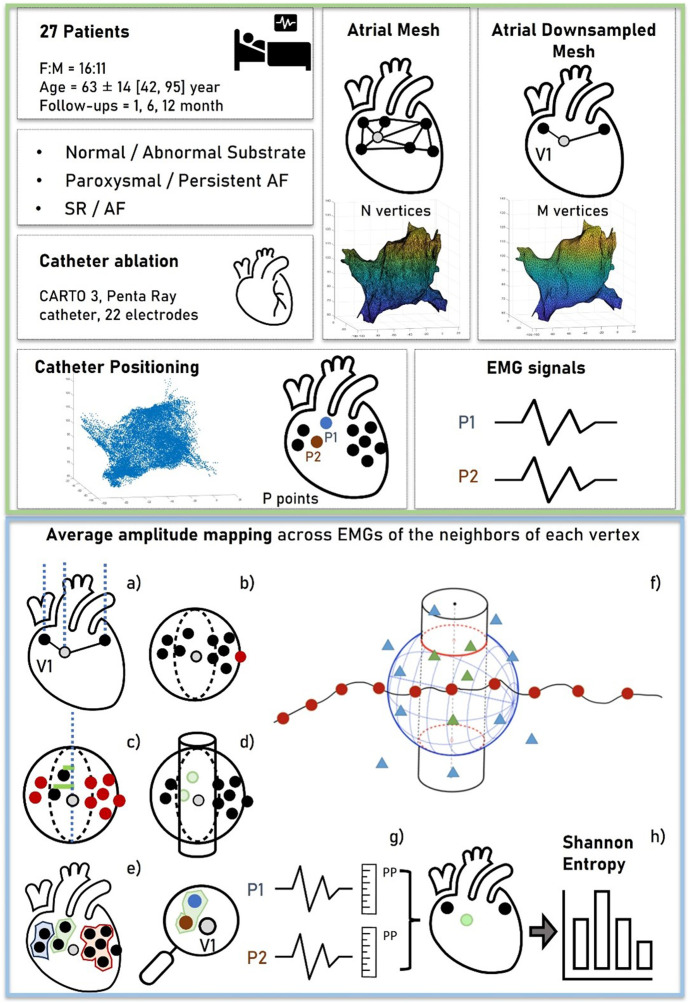
Workflow of data acquisition and processing. The green box (top) represents the data acquisition. For each patient, a cloud of points representing the 3D locations of the EGM signals was collected. An atrial mesh was computed and processed by several steps to associate points and signals with the vertices, as represented in the blue box (bottom). **(A)** The normal of the vertex was outlined and represented. **(B)** The sphere with center in the vertex was used to exclude the farthest points. **(C,D)** The additional criterion based on the distance between the vertex and the points was represented to obtain the neighboring area (a cylinder), outlined in **(E). (F)** Red points ideally represent the vertices of the mesh, the triangles represent the locations of the acquired EGM signals, and the resulting associated points are depicted by green triangles. From these, the average peak-to-peak (PP) of the EGMs was computed as shown in **(G)**, obtaining a voltage map of the mesh on which the Shannon entropy was computed **(H)**.

During LA mapping, bipolar signals were routinely acquired for 2.5 s and stored along with the 3D location of the mapping catheter at the time of recording. The set of locations visited by the mapping electrodes (mean = 13,000 ± 4,242) was presented as a cloud of points in space. An atrial mesh was also derived using signals <3.5 mV of amplitude, which was considered the noise threshold (more details on its creation are given in Section 2.3.1). The main steps of data acquisition and processing are summarized in green and blue boxes in Figure 1, respectively.

#### 2.3.1 Mesh creation and downsampling

The resolution of LA mesh obtained by CARTO 3 varied among patients, and depending on the number of vertices and triangles acquired during mapping. In order to compare results among different patients, the mesh was downsampled, setting a value of 5,000 vertices per patient with a tolerance of ± 100 vertices. In those cases with the number of vertices lower than 5,000, upsampling was performed. The resampling process was carried out through the open-source software “Meshtool” (Neic et al., 2020). This tool reduces mesh resolution by initially collapsing all edges smaller than a specified minimum value. Subsequently, it divides edges larger than the maximum edge length. The triangles are defined according to the Delaunay triangulation.

#### 2.3.2 Voltage map determination

After mesh homogenization, a surrogate of entropy measure was derived from each EGM associated with the vertex of the mesh. To accomplish this objective, (I) the vector corresponding to the normal of the mesh at each vertex (outward direction aligned with the mesh’s conformation and passing through the vertex) was determined (Figure 1A); (II) a sphere with the center located at the vertex was drawn, and only EGMs collected at positions inside the sphere were considered “candidates” associated with the vertex (Figure 1B); (III) the radius of the sphere was chosen empirically (8 mm) to encompass positions that, due to atrial movements, fell either below or above the vertex along the normal; and (IV) among the candidate EGMs, only those within a predefined distance from the normal were selected (Figure 1C). This further criterion was conceived to consider possible movements of the atria and account for deformations of the atrial wall during the electrophysiological procedure. From this concept, a cylinder was created by the summation of neighboring points, with its axis normal to the internal surface of the mesh and a radius of 2 mm (empirically determined considering the average distance between vertices) (Figure 1D).

Upon the completion of the neighboring points’ identification phase, it was possible to define a set of vertices $V=\left\{1,\ldots ,v\right\}$ , which were those of the mesh, associated with a set of neighbors $N_{v}=\left\{n:n\text{ neighbor of }v\right\}\;v\in V$ . Hence, the neighboring points related to a vertex v were defined as those within the sphere and having a distance from the normal vector that was less than the average distance between vertices (2 mm). This set of points $N_{v}$ had cardinality $N_{v}=\#N_{v}$ (indicated by the same colors in Figure 1, panel e, and green triangles in Figure 1F).

For each vertex v of the mesh, the range of voltage amplitudes (through the peak-to-peak amplitude formulation $\max\left(x_{n}\right)-\min\left(x_{n}\right)$ ) was computed on the EGMs acquired at each selected neighboring point, where *x*_*n*_ represents the *n*th 2.5-s EGM associated with the *n* point. The average of the amplitude range of all neighbors, i.e., $\frac{1}{N_{v}}\sum_{n=1}^{N_{v}}\left(\max\left(x_{n}\right)-\min\left(x_{n}\right)\right)$ , was then taken as the voltage range in v and used to determine the voltage map (Figure 1G).

#### 2.3.3 Entropy computation

Discrete entropy H was computed from the probability distribution of the amplitude range values associated with each vertex of the mesh, represented by histograms (Figure 1H). The resulting formulation expressed as a sum over all bins of the histogram was

$$H=-\sum_{i=1}^{I}p_{i}\log\left(\frac{p_{i}}{w}\right),$$

where w was the width of each bin (100 a.u. corresponding to 0.3 mv, with a conversion factor of 0.003 mV/a. u.) and $I=\left\{1,\ldots ,i\right\}$ was the set comprising all bins of the histogram with cardinality I=#I . Summarizing the methodology, it was possible to define the probability of the i th bin as follows (where $w_{0}$ is a constant value):

$$p_{i}=\frac{\#\left\{v:w_{0}+w\left(i-1\right)\leq \frac{1}{N_{v}}\sum_{n\in N_{v}}\left(\max\left(x_{n}\right)-\min\left(x_{n}\right)\right)\;<\;w_{0}+wi,v\in V\;\right\}}{\#V}.$$

The bin width w = 0.3 mV was selected to improve the capability to distinguish between scar tissue and noise. Voltage amplitude levels below 0.5 mV are widely recognized as indicative of significant fibrosis or scar tissue within the cardiac substrate. Notably, a very recent study identified 0.3 mV as the cutoff voltage that effectively enhanced the identification of scar tissue from the surrounding healthy myocardium (Roca-Luque et al., 2023).

### 2.4 Statistical analysis

All quantitative variables displayed a normal distribution, which was verified empirically by the visual inspection of the histogram. They were reported as the mean ± standard deviation and compared using a one-tailed Student’s t-test. *p* < 0.05 was considered significant. To evaluate the degree of agreement or comparability between continuous variables of measurements obtained from the calculation of entropy on the first mesh and the downsampled mesh, a Bland–Altman analysis was performed. Discrimination, i.e., the model’s ability to differentiate between patients with abnormal and normal LA, was examined using the area under the receiver operating characteristic (AUROC) curve. AUROC analysis was also performed to calculate cutoff values, sensitivity, and specificity. Finally, the cutoff points were calculated by obtaining the best Youden index (sensitivity + specificity −1). All statistical analyses were performed by two blinded researchers, and then, the results were compared for reproducibility using k-statistics. Data were analyzed using R software version 3.6.2 (R Foundation for Statistical Computing, Vienna, Austria).

## 3 Results

### 3.1 Study population and procedure characteristics

The main characteristics of the study population (mean age, 63 ± 14 years; 41% male) are summarized in Table 1. Overall, 16 patients (59%) suffered from paAF, while the remaining patients were affected by the persistent type. A normal LA substrate was reported in 15 patients with paAF (94%) and 2 patients with peAF (18%). The EAM was obtained during SR in 13 patients (48%), and the average number of collected EGMs per map was 3,029 ± 702 points. The mean skin-to-skin time was 56.3 ± 7.2 min, with a mean mapping time of 15.6 ± 3.4 min. Procedural success was achieved in all cases without procedural complications. After discharge, patients underwent follow-up for a mean time of 15 ± 2 months. At 1 year, two cases of AF recurrence were reported among peAF patients.

**TABLE 1:** Characteristics of the study population.

Clinical characteristic	
No. of patients	27
Age	63 ± 14
Male sex	11 (41%)
Former smoking	6 (22%)
Paroxysmal AF	16 (59%)
Hypertension	10 (37%)
Dyslipidemia	6 (22%)
Diabetes mellitus	3 (11%)
AAD	14 (52%)
Procedural characteristics	
SR during mapping	13 (48%)
Normal LA substrate at voltage map	17 (63%)
LVA, cm^2^ (mean, minimum, and maximum) Paroxysmal AF Persistent AF LVA/LA volume, % Paroxysmal AF Persistent AF	2 (0–3) 14 (8–21) <2 23 (16–27)
Skin-to-skin time, min	62.3 ± 6.9
Mapping time, min	15.6 ± 3.4
No. of acquired points	2029 ± 602
RF time, min	31.3 ± 5.3
AF recurrence during follow up (%)	2 (7%)

AAD, antiarrhythmic drug; AF, atrial fibrillation; LA, left atrial; RF, radiofrequency; LVA, low-voltage area; SR, sinus rhythm.

### 3.2 LASE values

**FIGURE 2:**
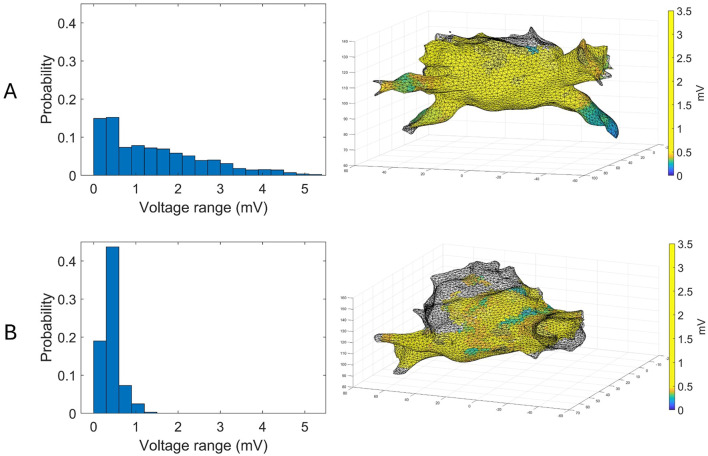
Examples of voltage maps superimposed to their corresponding atrial mesh (right) and their distributions (left) for a patient with paAF **(A)** and peAF **(B)**.

**FIGURE 3:**
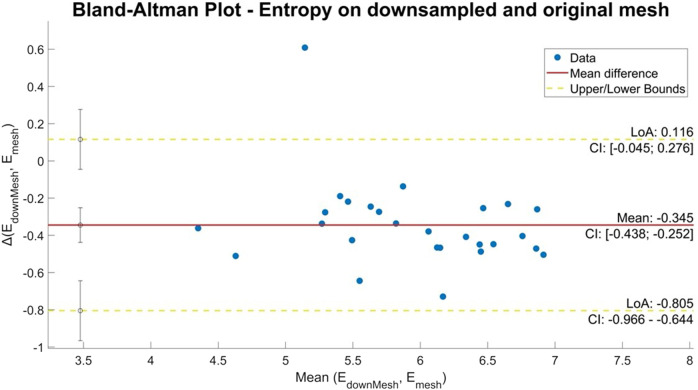
Bland–Altman plot of entropy values obtained from the original and downsampled meshes. The horizontal axis shows the average of the two entropy values and the vertical axis the difference between the two values.

**FIGURE 4:**
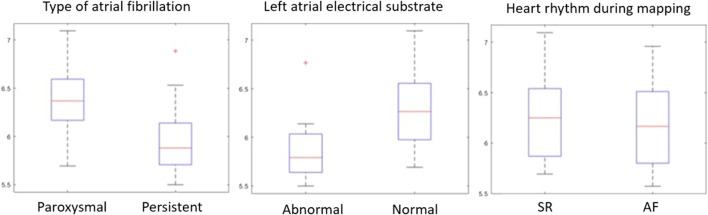
Distributions of LASE according to patients’ characteristics.

**FIGURE 5:**
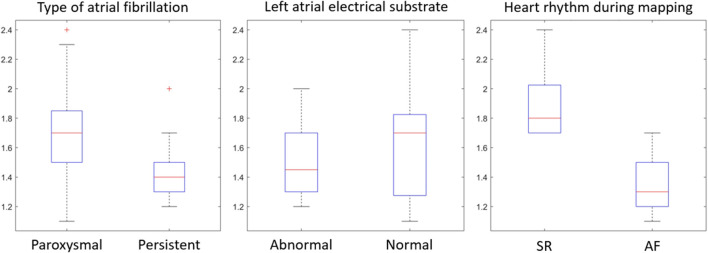
Distributions of EGM according to patients’ characteristics.

**FIGURE 6:**
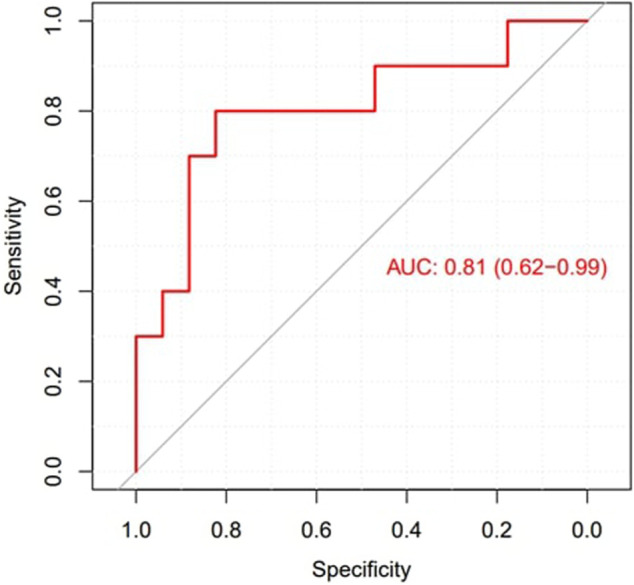
ROC curve to explore the diagnostic ability of LASE in identifying LA abnormal substrates.

The mean entropy calculated from the original mesh was 5.87 ± 0.47. After mesh downsampling, the mean entropy was 6.27 ± 0.53. Figure 2 shows an example of the distribution of two patients with high and low entropy values, while the values computed for the entire dataset are given in Table 2. The Bland–Altman analysis was performed to evaluate the degree of agreement between measurements obtained from the calculation of entropy on the first mesh and on the downsampled mesh (Figure 3). The average of the differences was −0.35, a bias value related to the calculation of the entropy in the original mesh. The measurements were, however, largely in agreement, with all but one case falling within the 95% confidence interval of the mean (between −0.81 and 0.12) and most within a range ±0.2 with respect to the mean (23 cases out of 27). First, all samples and related histograms of entropy distribution were qualitatively examined. Statistical tests were carried out on the entropy values relating to the type of substrate, the type of AF, and the heart rhythm during the procedure. Entropy values significantly differed between patients with paAF and peAF (6.45 ± 0.41 *versus* 5.87 ± 0.53; *p* = 0.028), as well as in patients with normal and abnormal LA substrates (6.42 ± 0.42 *versus* 5.87 ± 0.56; *p* = 0.043). Finally, it is worth noting that the entropy values were independent of the presence of SR or AF during the procedure (6.33 ± 0.41 *versus* 6.11 ± 0.63; *p* = 0.619). LASE and EGM distributions according to patients’ characteristics are given in Figures 4, 5, respectively. Compared to LASE, the EGM boxplot analysis did not distinguish between paAF and peAF (*p* = 0.053) or between normal and abnormal LA substrates (*p* = 0.22). Moreover, the basal rhythm significantly influenced the EGM values (*p* = <0.001). The accordance between the LASE and abnormal substrate identified in the voltage map was confirmed using the ROC curve (AUC: 0.81; CI: 0.62–0.99) (Figure 6). The cutoff with the best accuracy, according to the Youden index, was estimated at 6.06, demonstrating a sensitivity of 80% and a specificity of 80%. All results were compared for reproducibility, with a k-statistic of 0.82.

**TABLE 2:** Substrates, AF types, and derived LASE for each patient in the study.

Patient ID	Substrate	AF type	Basal rhythm	Entropy from the original mesh	LASE	AF recurrence
1	Normal	Paroxysmal	SR	6.206	6.693	No
2	Normal	Paroxysmal	SR	6.135	6.543	No
3	Normal	Paroxysmal	SR	5.804	6.533	No
4	Abnormal	Persistent	AF	4.374	4.885	Yes
5	Normal	Paroxysmal	AF	5.892	6.358	No
6	Abnormal	Persistent	AF	4.170	4.532	No
7	Normal	Paroxysmal	AF	6.319	6.767	No
8	Normal	Paroxysmal	AF	6.737	6.998	No
9	Abnormal	Persistent	AF	5.557	5.831	No
10	Normal	Paroxysmal	SR	5.803	5.940	No
11	Normal	Persistent	AF	5.509	5.755	No
12	Abnormal	Persistent	SR	5.652	5.988	No
13	Abnormal	Persistent	AF	5.448	4.839	Yes
14	Normal	Paroxysmal	AF	6.535	6.767	No
15	Normal	Paroxysmal	SR	6.556	6.960	No
16	Abnormal	Persistent	AF	5.281	5.707	No
17	Normal	Paroxysmal	SR	5.872	6.251	No
18	Normal	Paroxysmal	SR	5.914	6.380	No
19	Normal	Persistent	AF	5.101	5.439	No
20	Normal	Paroxysmal	SR	6.662	7.167	No
21	Normal	Paroxysmal	SR	6.216	6.665	No
22	Abnormal	Persistent	AF	5.156	5.432	No
23	Normal	Paroxysmal	SR	6.625	7.096	No
24	Normal	Paroxysmal	SR	6.340	6.594	No
25	Abnormal	Persistent	AF	5.354	5.573	No
26	Abnormal	Paroxysmal	SR	5.228	5.872	No
27	Abnormal	Persistent	AF	5.310	5.500	No

At follow-up, patients with AF recurrence were characterized by the lowest LASE.

## 4 Discussion

To the best of our knowledge, this is the first study using spatial entropy for further characterization of the whole LA electrical substrate in patients affected by AF, independent of common EGM thresholds of the bipolar map. The main findings of the study are as follows: (i) the LASE significantly differentiated paAF (higher LASE) from peAF (lower LASE), as well as the presence of normal (higher LASE) or abnormal (lower LASE) LA substrate; (ii) LASE was independent of the heart rhythm (SR vs. AF) during map acquisition; (iii) LASE was in accordance with the EAM map with a high sensibility and specificity.

### 4.1 Models of entropy translated to AF

According to information theory (Shannon, 1948; Rivolta, 2024), entropy provides an estimate of the number of bits required to encode a random event. If the probability of a certain event is high, its informative content tends to 0. Therefore, if one event in the distribution is nearly certain to happen, only one bit is necessary. Conversely, if the probability of the event is low, the number of bits required for encoding it will be high (Ribeiro et al., 2021). Subsequently, the higher the probability of a group of events occurring with the same frequency, the higher the value of entropy. On the contrary, if the probability of the events occurring with the same frequency is low, entropy will be low.

Numerous studies used entropy to address multiple tasks related to physiological signals, including cardiac data recording (Rivolta, 2024; Ganesan et al., 2014; Manikandan and Soman, 2012). Applying this concept to the electrical activity of LA, if the range of atrial voltage amplitudes is uniform in every region, its entropy will be high. This occurs because the probability of encountering a specific voltage is similar across all locations explored during mapping. Conversely, if the electrical activity is not uniformly distributed due to the presence of fibrosis or LVAs, resulting in a skewed probability distribution, entropy will be lower.

In the context of the present study, the histogram pattern in panel A of Figure 2 (wider but more homogeneous distribution of probability) is representative (with statistical significance) of a typical profile for a normal substrate and paroxysmal AF, and it corresponds to the presence of similar amplitudes in different locations. Contrarily, panel B of Figure 2 is typical of an abnormal substrate, with different values of voltage across the atrial tissue. Intriguingly, the probability of voltage amplitude distribution seems independent of the rhythm present at the time of mapping. Therefore, it remains significantly higher in patients with normal substrate even if the data acquisition was performed during AF, which usually represents an important limitation for bipolar mapping. This is probably because the data acquisition of the explored area was based on a sample of voltage amplitude distribution and not only on the single voltage value recorded at a certain time. For this reason, LASE may overcome some well-known limitations of conventional bipolar mapping, such as arbitrary LVA thresholds, basal rhythm, and the direction of the wavefront relative to the orientation of the mapping catheter electrode (de Bakker, 2019; Yamaguchi et al., 2019).

### 4.2 LASE computation

As the variability observed in the original meshes across different patients may influence the comparability of the findings, we downsampled the mesh by reducing the vertex count, providing more uniformity. This reduction aimed to standardize the datasets for inter-patient comparison and provide faster computation and processing. This process did not lead to the loss of information, as confirmed by the results of the comparative analysis.

The criteria for selecting candidate EGMs considered their proximity to the normal to the inner surface of the LA. The aim was to address potential issues related to atrial movements and wall deformations during the electrophysiological procedure. This method added a valuable refinement layer based on the average distance between vertices.

The definition of LVA was historically arbitrarily defined, with large differences among studies (Junarta et al., 2022). Previously, LVAs were defined by at least three points of low voltage acquired using the ablation catheter, which is typically equipped with only two dipoles. This method has provided conflicting results regarding the long-term effectiveness of adding ablation of these regions to conventional PV ablation (Yang et al., 2022; Kircher et al., 2018). However, these studies confirmed the beneficial role of voltage maps in identifying patients with peAF and normal LA substrates, who may achieve excellent rhythm control with PV isolation alone. The proposed workflow enables the assessment of the SE across the entire atrium based on voltage maps. Alternative approaches based on localized evaluations within specific areas (e.g., left/right pulmonary veins) or leveraging surrogate data through bootstrapping techniques may offer a wider view of the entropy distribution and its variability.

The relevance of entropy within the electrophysiological framework supports its wider application, particularly in the field of AF, which is, by definition, the most chaotic rhythm (Nicolet et al., 2019). In addition, it may be interesting to explore other concepts of entropy applied to voltage maps, such as dispersion entropy (Rostaghi and Azami, 2016) and fuzzy entropy (Chen et al., 2007). In this regard, additional characteristics of the EGM signals different from voltage amplitude or SE of the time series may be analyzed.

### 4.3 Clinical implications

To date, several mapping tools have been developed to improve our understanding of AF, such as CFAE (Hunter et al., 2011) and CARTOFINDER (Honarbakhsh et al., 2018), which aim to detect high-rate focal or rotational activity. However, these tools have failed to improve long-term outcomes and are dependent on the presence of FA during mapping (Narayan and John, 2024). Recently, omnipolar and multipolar mapping techniques have been introduced to overcome the well-known limitations of bipolar mapping, providing optimistic results in substrate mapping and identifying gaps in the ablation lines around the pulmonary veins (Butcher et al., 2023). Finally, the most recent functional mapping method was proposed to analyze, during SR, the response to short-coupled atrial extrastimuli to identify areas of hidden slow conduction within the LA (Silva Garcia et al., 2023). In our study, we investigate the relationship between LASE and the clinical patterns of AF, as well as LASE and LA substrate. The clinical behavior of AF and the presence of LVAs in the voltage map are widely accepted surrogate markers of the degree of atrial structural and electrical remodeling and are associated with a worse outcome after ablation (Starek et al., 2023). However, these factors lack accuracy in predicting AF recurrence patient by patient. This is partially explained by the fact that AF is a complex arrhythmia without a full understanding of its underlying mechanisms. Therefore, performing CA with the EAM system is not only a therapeutic procedure but also a diagnostic opportunity, providing useful insights into the degree of the disease.

Since high-density substrate mapping of the LA encounters several limitations, LASE was used to increase its diagnostic accuracy. Previous studies used entropy to improve the diagnostic power of already existing tools designed to identify such functional electrical anomalies underlying arrhythmia sustainability (Ng et al., 2010). However, all these methods reported contrasting results in terms of effectiveness and capability, and their use is not yet suggested by the guidelines. Our research aimed to identify a novel indicator capable of working synergistically with the EAM system to provide useful information about the underlying AF mechanism and risk recurrence at follow-up. The LASE diagnostic potential was significant, reporting an AUC of 0.81 with 80% sensibility and specificity in predicting abnormal LA substrates. Of note, patients with early recurrence of AF at follow-up were characterized by very low LASE. This finding may be proof for further studies exploring the utility of this tool in predicting post-CA arrhythmia recurrences, compared to conventional parameters that are routinely considered.

Ultimately, LASE demonstrated to be both a feasible and reliable measure, capable of being calculated from a standard bipolar map at any given moment after retrospective exporting and processing. This might facilitate eventual comparative analysis of ablations within the same patient or across different patients. Consequently, this novel tool can be easily translated into the clinical setting of CA, assisting a tailored substrate-based strategy.

### 4.4 Study limitations

The small sample is a major limitation to the study, warranting the need for larger validation studies to confirm our findings. Since all EAMs were produced using the CARTO 3 system, LASE remains, at present, a tool developed for a single-mapping system and, therefore, influenced by all its inherent limitations, limiting its applicability to other mapping technologies. The accuracy and reliability of the results should be independent of the number and density of acquired points. In this study, we adhered to a general practice of utilizing maps comprising a minimum of 1,000 points to ensure accuracy and reliability. However, the selection of the threshold was arbitrary, and the impact of such map density on LASE remains uncertain. This hypothesis necessitates further confirmation. Finally, the specific supplementary insights provided by LASE with respect to standard voltage mapping and its power in predicting AF recurrences after CA are still unresolved questions. Regional LASE assessed at different parts of LA may potentially guide the ablation strategy, particularly in non-exhaustive bipolar maps.

## 5 Conclusion

A continuum of electrophysiological abnormalities characterizes the progression of the electrical atrial substrate and AF recurrence after CA. In our study, LASE was correlated with the clinical classification of AF and EAM of LA, independent of basal rhythm.

## Data Availability

The raw data supporting the conclusion of this article will be made available by the authors, without undue reservation.

## References

[B1] Arbelo E. Brugada J. Hindricks G. Maggioni A. P. Tavazzi L. Vardas P. (2014). The atrial fibrillation ablation pilot study: a European Survey on Methodology and results of catheter ablation for atrial fibrillation conducted by the European Heart Rhythm Association. Eur. Heart J. 35, 1466–1478. 10.1093/eurheartj/ehu001 24487524

[B2] Berruezo A. Tamborero D. Mont L. Benito B. Tolosana J. M. Sitges M. (2007). Pre-procedural predictors of atrial fibrillation recurrence after circumferential pulmonary vein ablation. Eur. Heart J. 28, 836–841. 10.1093/eurheartj/ehm027 17395676

[B3] Butcher C. Roney C. Wharmby A. Ahluwalia N. Chow A. Lambiase P. D. (2023). In atrial fibrillation, omnipolar voltage maps more accurately delineate scar than bipolar voltage maps. JACC Clin. Electrophysiol. 9, 1500–1512. 10.1016/j.jacep.2023.03.010 37204357

[B4] Chen W. Wang Z. Xie H. Yu W. (2007). Characterization of surface EMG signal based on Fuzzy entropy. IEEE Trans. Neural Syst. Rehabilitation Eng. 15, 266–272. 10.1109/TNSRE.2007.897025 17601197

[B5] Chugh S. S. Havmoeller R. Narayanan K. Singh D. Rienstra M. Benjamin E. J. (2014). Worldwide epidemiology of atrial fibrillation: a global burden of disease 2010 study. Circulation 129, 837–847. 10.1161/circulationaha.113.005119 24345399 PMC4151302

[B6] de Bakker J. M. (2019). Electrogram recording and analyzing techniques to optimize selection of target sites for ablation of cardiac arrhythmias. Pacing Clin. Electrophysiol. 42, 1503–1516. 10.1111/pace.13817 31609005 PMC6916598

[B7] Ganesan A. N. Kuklik P. Gharaviri A. Brooks A. Chapman D. Lau D. H. (2014). Origin and characteristics of high shannon entropy at the pivot of locally stable rotors: insights from computational simulation. PLoS One 9, e110662. 10.1371/journal.pone.0110662 25401331 PMC4234245

[B8] Ganesan A. N. Kuklik P. Lau D. H. Brooks A. G. Baumert M. Lim W. W. (2013). Bipolar electrogram shannon entropy at sites of rotational activation: implications for ablation of atrial fibrillation. Circ. Arrhythm. Electrophysiol. 6, 48–57. 10.1161/circep.112.976654 23264437

[B9] Honarbakhsh S. Schilling R. J. Dhillon G. Ullah W. Keating E. Providencia R. (2018). A novel mapping system for panoramic mapping of the left atrium: application to detect and characterize localized sources maintaining atrial fibrillation. JACC Clin. Electrophysiol. 4, 124–134. 10.1016/j.jacep.2017.09.177 29387810 PMC5777816

[B10] Hunter R. J. Diab I. Tayebjee M. Richmond L. Sporton S. Earley M. J. (2011). Characterization of fractionated atrial electrograms critical for maintenance of atrial fibrillation: a randomized, controlled trial of ablation strategies (the CFAE AF trial). Circulation Arrhythmia Electrophysiol. 4, 622–629. 10.1161/CIRCEP.111.962928 21844156

[B11] Hwang M. Song J. S. Lee Y. S. Li C. Shim E. B. Pak H. N. (2016). Electrophysiological rotor ablation in in-silico modeling of atrial fibrillation: comparisons with dominant frequency, shannon entropy, and phase singularity. PLoS One 11, e0149695. 10.1371/journal.pone.0149695 26909492 PMC4766081

[B12] Jadidi A. S. Lehrmann H. Keyl C. Sorrel J. Markstein V. Minners J. (2016). Response to letter regarding article, ablation of persistent atrial fibrillation targeting low-voltage areas with selective activation characteristics. Circ. Arrhythm. Electrophysiol. 9, e004312. 10.1161/CIRCEP.116.004312 27390213

[B13] Junarta J. Siddiqui M. U. Riley J. M. Dikdan S. J. Patel A. Frisch D. R. (2022). Low-voltage area substrate modification for atrial fibrillation ablation: a systematic review and meta-analysis of clinical trials. EP Eur. 24, 1585–1598. 10.1093/europace/euac089 35696286

[B14] Kircher S. Arya A. Altmann D. Rolf S. Bollmann A. Sommer P. (2018). Individually tailored vs. standardized substrate modification during radiofrequency catheter ablation for atrial fibrillation: a randomized study. Europace 20, 1766–1775. 10.1093/europace/eux310 29177475

[B15] Li K. Xu C. Zhu X. Wang X. Ye P. Jiang W. (2023). Multi-centre, prospective randomized comparison of three different substrate ablation strategies for persistent atrial fibrillation. EP Eur. 25, euad090. 10.1093/europace/euad090 PMC10228617 37050858

[B16] Manikandan M. S. Soman K. P. (2012). A novel method for detecting R-peaks in electrocardiogram (ECG) signal. Biomed. Signal Process. Control 7, 118–128. 10.1016/j.bspc.2011.03.004

[B17] Marcon L. Bergonti M. Spera F. Saenen J. Huybrechts W. Miljoen H. (2023). Dynamic changes of left atrial substrate over time following pulmonary vein isolation: the Progress-AF study. Europace 25, euad299. 10.1093/europace/euad299 37787610 PMC10629715

[B18] Mateos D. M. Guevara Erra R. Wennberg R. Perez Velazquez J. L. (2018). Measures of entropy and complexity in altered states of consciousness. Cogn. Neurodyn 12, 73–84. 10.1007/s11571-017-9459-8 29435088 PMC5801282

[B19] Rivolta M. W. (2024). Information theory and fetal heart rate variability analysis. in Innovative technologies and signal processing in perinatal medicine. Switzerland: Springer, 171–188. 10.1007/978-3-031-32625-7_9

[B20] Narayan S. M. John R. M. (2024). Advanced electroanatomic mapping: current and emerging approaches. Curr. Treat. Options Cardiovasc. Med. 26, 69–91. 10.1007/s11936-024-01034-6 PMC12525732 41104347

[B21] Neic A. Gsell M. A. F. Karabelas E. Prassl A. J. Plank G. (2020). Automating image-based mesh generation and manipulation tasks in cardiac modeling workflows using Meshtool. SoftwareX 11, 100454. 10.1016/j.softx.2020.100454 32607406 PMC7326605

[B22] Ng J. Borodyanskiy A. I. Chang E. T. Villuendas R. Dibs S. Kadish A. H. (2010). Measuring the complexity of atrial fibrillation electrograms. J. Cardiovasc Electrophysiol. 21, 649–655. 10.1111/j.1540-8167.2009.01695.x 20132398

[B23] Nicolet J. Schlotthauer G. Restrepo Rinckoar J. (2019). Classification of intracavitary electrograms in atrial fibrillation using information and complexity measures. Biomed. Signal Process. Control 57, 101753. 10.1016/j.bspc.2019.101753

[B24] Parameswaran R. Al-Kaisey A. M. Kalman J. M. (2021). Catheter ablation for atrial fibrillation: current indications and evolving technologies. Nat. Rev. Cardiol. 18, 210–225. 10.1038/s41569-020-00451-x 33051613

[B25] Pratola C. Baldo E. Notarstefano P. Toselli T. Ferrari R. (2008). Radiofrequency ablation of atrial fibrillation: is the persistence of all intraprocedural targets necessary for long-term maintenance of sinus rhythm? Circulation 117, 136–143. 10.1161/circulationaha.106.678789 18086927

[B26] Proietti R. Santangeli P. Di Biase L. Joza J. Bernier M. L. Wang Y. (2014). Comparative effectiveness of wide antral versus ostial pulmonary vein isolation: a systematic review and meta-analysis. Circ. Arrhythm. Electrophysiol. 7, 39–45. 10.1161/circep.113.000922 24385448

[B27] Ribeiro M. Henriques T. Castro L. Souto A. Antunes L. Costa-Santos C. (2021). The entropy universe. Entropy 23, 222. 10.3390/e23020222 33670121 PMC7916845

[B28] Roca-Luque I. Zaraket F. Garre P. Sanchez-Somonte P. Quinto L. Borras R. (2023). Accuracy of standard bipolar amplitude voltage thresholds to identify late potential channels in ventricular tachycardia ablation. J. Interv. Card. Electrophysiol. 66, 15–25. 10.1007/s10840-022-01148-6 35195814 PMC9931851

[B29] Rodríguez-Mañero M. Valderrábano M. Baluja A. Kreidieh O. Martínez-Sande J. L. García-Seara J. (2018). Validating left atrial low voltage areas during atrial fibrillation and atrial flutter using multielectrode automated electroanatomic mapping. JACC Clin. Electrophysiol. 4, 1541–1552. 10.1016/j.jacep.2018.08.015 30573117

[B30] Romero J. Polanco D. Gabr M. Alviz I. Diaz J. C. Briceno D. (2023). Posterior wall electrical isolation in patients undergoing catheter ablation for paroxysmal and nonparoxysmal atrial fibrillation. JACC Clin. Electrophysiol. 9, 583–585. 10.1016/j.jacep.2022.11.007 36752475

[B31] Rostaghi M. Azami H. (2016). Dispersion entropy: a measure for time series analysis. IEEE Signal Process. Lett. 23, 610–614. 10.1109/LSP.2016.2542881

[B32] Shannon C. E. A. (1948). A mathematical theory of communication. Bell Syst. Tech. J. 27, 379–423. 10.1002/j.1538-7305.1948.tb01338.x

[B33] Shaw D. Charles H. D. (1983). Entropy and information: a multidisciplinary overview. J. Am. Soc. Inf. Sci. 34, 67–74. 10.1002/asi.4630340110

[B34] Silva Garcia E. Lobo-Torres I. Fernández-Armenta J. Penela D. Fernandez-Garcia M. Gomez-Lopez A. (2023). Functional mapping to reveal slow conduction and substrate progression in atrial fibrillation. Europace 25, euad246. 10.1093/europace/euad246 37961921 PMC10644200

[B35] Starek Z. Di Cori A. Betts T. R. Clerici G. Gras D. Lyan E. (2023). Baseline left atrial low-voltage area predicts recurrence after pulmonary vein isolation: WAVE-MAP AF results. Europace 25, euad194. 10.1093/europace/euad194 37470443 PMC10410193

[B36] Tzeis S. Gerstenfeld E. P. Kalman J. Saad E. B. Sepehri Shamloo A. Andrade J. G. (2024). 2024 European heart rhythm association/heart rhythm society/asia pacific heart rhythm society/Latin American heart rhythm society expert consensus statement on catheter and surgical ablation of atrial fibrillation. EP Eur. 26, euae043. 10.1093/europace/euae043 38597857

[B37] Vlachos K. Efremidis M. Letsas K. P. Bazoukis G. Martin R. Kalafateli M. (2017). Low-voltage areas detected by high-density electroanatomical mapping predict recurrence after ablation for paroxysmal atrial fibrillation. J. Cardiovasc Electrophysiol. 28, 1393–1402. 10.1111/jce.13321 28884923

[B38] Wu K. C. Chrispin J. (2022). More than meets the eye: cardiac magnetic resonance image entropy and ventricular arrhythmia risk prediction. JACC Cardiovasc Imaging 15, 793–795. 10.1016/j.jcmg.2022.01.012 35331659 PMC9169813

[B39] Yamaguchi T. Fukui A. Node K. (2019). Bipolar voltage mapping for the evaluation of atrial substrate: can we overcome the challenge of directionality? J. Atr. Fibrillation 11, 2116. 10.4022/jafib.2116 31139298 PMC6533827

[B40] Yang G. Zheng L. Jiang C. Fan J. Liu X. Zhan X. (2022) Circumferential pulmonary vein isolation plus low-voltage area modification in persistent atrial fibrillation: the STABLE-SR-II trial. JACC Clin. Electrophysiol. 8, 882–891. 10.1016/j.jacep.2022.03.012 35863814

